# Spina Bifida

**Published:** 1861

**Authors:** 


### SPINA BIFIDA.

Dr. Douglas, of this city, injected a spina-bifida with solution of iodine, on a male infant seven weeks old. The plan adopted was that proposed and used by Prof. Brainard, of Chicago.

The lesion in this case is lumbo-sacral, and nearly three inches in diameter; its complications are paralysis and hydrocephalus. The child had thirteen convulsions within the four weeks preceding the operative measure, which was first practiced on the fourth of March. No convulsion or other unpleasant symptom followed the use of the injection.

Up to the 11th of March there were some improvements in the intensity of the symptoms which had previously existed. The tumor had now again refilled, and the protuberance of the anterior fontanelle was as great as before; the clonic spasm still persisted, and the extra pronation of the hands was little if any better. The infant had no convulsions after the first injection until this date. The tumor had now assumed its former dimensions.

The attending physician (Dr. Douglas) determined to perform the second operation on this date; (seven days elapsing from the first;) before injecting the tumor, moderate pressure upon it brought on a violent convulsion which lasted four or five minutes. It was now treated as before, and as soon as the tumor at the fontanelle showed signs of subsidence, the iodine injection was thrown into the lumbo-sacral sac. No unpleasant effects followed its immediate use.

From that period up to this date, March 25th, there has been a general and marked improvement in all the abnormal symptoms of the case. The extra pronation of the hands has ceased, the strabismus has disappeared, the cephalic region has not again been invaded with the excess of cerebro-spinal fluid, and the lumbo-sacral tumor is gradually diminishing in all its amplitudes. The head hangs more natural and erect, the tendency of falling back being unobserved except when the infant sleeps. The persistent tendency to clonic spasm still remains, and there is little if any amendment in the paralysis of the lower extremities.

The progress of the case thus far is satisfactory, and much better than to let them die without an effort to save. If Dr. Douglas saves (this very unpromising case in the beginning) his case, he will have accomplished what some of our best surgeons have failed in, and it will be the first successful case treated on this coast, and, under its present features, he has fair grounds of hope. We congratulate him on his success thus far. We shall watch this case with interest, and report its progress hereafter.—*Pacific Journal.*

*Remarks.*—The above case is interesting as furnishing additional proof of the safety of iodine injections. The details of the method employed are not given. At present I should close the opening into the spine, if possible, by pressing the sac into it as the fluid escaped; inject the sac with a stronger solution than would be proper where it is allowed to pass into the spinal canal and wash it out with distilled water or the fluid from the tumor. The complications in this case are such as to indicate malformation or extensive disease of the nervous centers and render the case unfavorable, as the cure of the spina-bifida can hardly be expected to remove the disease.

